# microRNA-30b/c inhibits non-small cell lung cancer cell proliferation by targeting Rab18

**DOI:** 10.1186/1471-2407-14-703

**Published:** 2014-09-24

**Authors:** Keng Zhong, Kun Chen, Lin Han, Bailing Li

**Affiliations:** Department of Cardiothoracic Surgery, Changhai Hospital affiliated to Second Military Medical University, Shanghai, 200433 China; Department of Respiratory Medicine, People’s Hospital of Tongren City, Guizhou, 554300 China

**Keywords:** miR-30b, miR-30c, Proliferation, Rab18, NSCLC

## Abstract

**Background:**

MicroRNAs (miRNAs) are small noncoding RNAs that post-transcriptional regulate gene expression in a variety of cancers. Increasing evidences indicate that miR-30 expression is down-regulated in numerous human cancers including non-small cell lung cancer (NSCLC) which hypothesizes that miR-30 may play an important role in tumorigenesis. The aim of this study was to investigate the target gene of miR-30 and its roles in tumor growth of NSCLC.

**Methods:**

Luciferase reporter assays were employed to validate regulation of a putative target of miR-30. The effect of miR-30 on endogenous levels of this target were subsequently confirmed via Western blot (WB). Quantitative reverse transcription-polymerase chain reaction (qRT-PCR) was performed to determine the expression level of miR-30 in NSCLC specimens and adjacent non-tumor tissues. MTT assays were conducted to explore the impact of miR-30 overexpression on the proliferation of human NSCLC cells.

**Results:**

Both miR-30b and miR-30c (miR-30b/c) were found having target site in same region of Rab18 mRNA. Luciferase assays using a reporter carrying a putative miR-30b/c target site in the coding DNA sequence (CDS) region of Rab18 revealed that miR-30b/c directly targeted Rab18. Overexpression of miR-30b/c led to down-regulation of Rab18 in A549 and H23 cells at protein levels but not mRNA levels. Down-regulation of miR-30b/c and up-regulation of Rab18 protein levels were detected in NSCLC specimens compared with adjacent non-tumor tissues. Overexpression of miR-30b/c suppressed NSCLC cells growth. Knockdown of Rab18 by siRNA significantly inhibited the proliferation of NSCLC cells.

**Conclusions:**

We demonstrated that miR-30b/c was down-regulated in NSCLC specimens compared with adjacent non-tumor tissues. miR-30b/c directly targeted and down-regulated Rab18 expression and inhibited NSCLC cells proliferation. These data indicated that miR-30b/c could serve as a tumor suppressor gene involved in NSCLC pathogenesis.

## Background

Lung cancer is the most common cause of cancer-associated deaths worldwide, especially for male [1]. NSCLC accounts for nearly 80% of lung cancers [2]. NSCLC population has grown quickly over the past five years in China [3]. Although surveillance and clinical treatment strategies have been improved, the 5-year survival of NSCLC patients after curative resection is reported to be only 30–60% [4]. Therefore, elucidating the potential mechanism that mediate the initiation and progression of NSCLC is urgent and of great interest.

miRNAs are a class of small non-coding RNAs which plays an important role in post-transcriptional regulation in various biological processes. Mechanistically, miRNAs bind to their target mRNAs and cause translation to be blocked or mRNA degradation [5, 6]. Accumulating evidences have suggested that miRNAs play diverse roles in tumorigenesis and cancer progression [7–10]. In recent years, miRNAs have received great attention in NSCLC research. Several deregulated miRNAs in NSCLCs such as miR-221, miR-222, miR-449a, miR-21, miR-205, miR-10b, miR-143 and miR-181a have been shown to regulate cell growth, apoptosis, migration and invasion [11–16]. These findings indicate that deregulation of miRNA expression may be associated with tumorigenesis of NSCLCs.

miR-30 is significantly down-regulated in several cancers, including breast cancer [17], malignant peripheral nerve sheath tumors [18], glioma [19], and lung cancer [20]. As the down-regulation of miR-30 is related to a number of cancers, it has been hypothesized that miR-30 may play an important role in tumorigenesis and tumor development. However, the function of miR-30 especially in NSCLC remains unclear.

In our study, we showed that Rab18 were identified as direct functional targets of miR-30b/c in NSCLC cells and miR-30b/c was down-regulated in NSCLC tissues compared to adjacent non-tumor tissues. Furthermore, ectopic overexpression of miR-30b/c blocked tumor cell proliferation *in vitro*. These data suggested that the reduced expression of miR-30b/c might facilitate the development of NSCLCs.

## Methods

### Specimens

In this study, 5 paired NSCLC and adjacent non-tumor specimens were collected from the Department of Respiratory Medicine, the Second Affiliated Hospital, Second Military Medical University (Shanghai, China). All tissue samples were flash-frozen in liquid nitrogen immediately after collection and stored at -80°C until use. The study protocol was approved by Shanghai Changzheng Hospital Ethical Committee. Informed consent was obtained from all patients. All clinic pathologic and biological data were available for those patients. Both tumor and non-tumor samples were confirmed by pathological examination. No patients received chemotherapy or radiotherapy prior to surgery.

### Cell culture

The human NSCLC cell lines A549 and H23 were purchased from ATCC. A549 cells were cultured in Ham’s F12K media (Invitrogen, Carlsbad, CA, USA) supplemented with 10% (vol/vol) fetal bovine serum (FBS) (Invitrogen, Carlsbad, CA, USA), H23 cells were cultured in Dulbecco’s Modified Eagle Medium (DMEM; Sigma-Aldrich, St. Louis, Mo., USA) supplemented with 10% (vol/vol) FBS, HEK293 cells were purchased from ATCC and grown were cultured in DMEM media containing 10% (vol/vol) FBS. Cells were maintained at 37°C in a humidified atmosphere with 5% CO_2_.

### RNA isolation and qRT-PCR

Total RNA was isolated from NSCLC tissues, adjacent non-tumor tissues and cell lines using Trizol according to the manufacturer’s instructions. qRT-PCR detection was performed as described previously [21]. U6 small RNA was used as an internal control for normalization and quantification of miR-30b/c expression. β-actin was used as an internal control for normalization and quantification of Rab18 expression. All primers were listed in Table 1.

**Table 1:** **All primers used in this study**

Name	Primer sequence
Rab18 (WT) F	5′-AAACTAGTTAACTCCCAGCTATTATAG-3′
Rab18 (WT) R	5′-GGAAGCTTTCTTCTTGTGACATCATAAAC-3′
Rab18 (MUT) F	5′-AAACTAGTTAACTCCCAGCTATTATAG-3′
Rab18 (MUT) R	5′-GGAAGCTTTCTTCTTCAGTGTAGAATATGTAA-3′
U6 RT	5′-GTCGTATCCAGTGCAGGGTCCGAGGTATTCGCACTGGATACGACTGGAAC-3′
U6 F	5′-AACGCTTCACGAATTTGCGT-3′
miR-30b RT	5′-GTCGTATCCAGTGCAGGGTCCGAGGTATTCGCACTGGATACGACAGCTGA-3′
miR-30b F	5′-ATCGCTGTAAACATCCTACAC-3′
miR-30c RT	5′-GTCGTATCCAGTGCAGGGTCCGAGGTATTCGCACTGGATACGACGCTGAG-3′
miR-30c F	5′-GCTTTGTAAACATCCTACACT-3′
Rab18 F	5′-TAAAGAGCCAGATAGGAA-3′
Rab18 R	5′-TCTATAATAGCTGGGAGTT-3′
β-actin F	5′-AGCAGCATCGCCCCAAAGTT-3′
β-actin R	5′-GGGCACGAAGGC TCATCATT-3′
miRNA Universal R	5′-GTGCAGGGTCCGAGGT-3′

*Abbreviations:* *RT* reverse-transcription primer, *F* forward primer, *R* reverse primer, *WT* wild type, *MUT* mutant.

### Western blot

The Western blot protocol was described previously [22]. Proteins were separated on a 12% SDS-PAGE gel and transferred onto a nitrocellulose membrane (Bio-Rad, Hercules, USA). The membrane was blocked with 5% non-fat milk and incubated with anti-Rab18 antibody (Proteintech Group) or anti-beta-actin antibody (Sigma, CA, USA). After being washed extensively, secondary antibody (Pierce, IL, USA) was added to the system. Immunoreactive protein bands were detected using an Odyssey Scanning system.

### Oligonucleotides transfection

RNA oligonucleotides were chemically synthesized and purified by Genepharma Co. Ltd., (Shanghai, China). Sequence of human miR-30b mimics was 5′- UGU AAA CAUC CUA CAC UCA GCU -3′ and human miR-30c mimics was 5′- UGU AAA CAU CCU ACA CUC UCA GC -3′. Negative control oligonucleotides for miRNA mimics was 5′-CAG UAC UUU UGU GUA GUA CAA-3′. The sequences of Rab18 siRNA was: 5′- GAA ACA UAC UGU ACA AGA ATT -3′ (sense) and 5′-UUC UUG UAC AGU AUG UUU CTT-3′ (antisense), Control siRNA was: 5′-UUC UCC GAA CGU GUC ACG UTT-3′ (sense), 5′-ACG UGA CAC GUU CGU AGA ATT-3′ (antisense). The transfections were performed with INTERFERin reagent (Polyplus-transfection). The final concentration of miRNA was 50 nM. The final concentration of siRNA was 20 nM.

### Luciferase assay

Luciferase reporter construct was made by cloning human Rab18 sequence containing the potential miR-30b/c binding site into pMIR-Report construct (Ambion, Austin, USA). Wild type (WT) or mutant (MUT) Rab18 fragment (from 501 to 562) was amplified by PCR using the primers in Table 1. All fragments were amplified and cloned into the luciferase repoter via *Spe*I and *Hind*III sites. Luciferase reporter assays were performed as previously [22], HEK293 cells were co-transfected with 50 nM miRNA mimics or negative control oligonucleotides, 100 ng of firefly luciferase reporter and 20 ng of pRL-TK (Promega, USA) using the JetPRIME reagent (Polyplus-transfection). Cells were collected 48 hours after last transfection and analyzed using Dual-Luciferase Reporter Assay System (Promega).

### MTT assay

The *in vitro* growth of NSCLC cells was measured using the MTT assay. 5000 cells were seeded into each well of 96-well plates and transfected with miRNA mimics or negative control oligonucleotides at a final concentration of 50 nM respectively. On the day of harvest, 100 μl of spent medium was replaced with an equal volume of fresh medium containing MTT 0.5 mg/ml. Plates were incubated at 37°C for 4 hrs, then the medium was replaced by 100 μl of DMSO (Sigma) and plates shaken at room temperature for 10 min. The absorbance was measured at 570 nm.

### Immunohistochemistry

Immunohistochemical (IHC) staining of samples was performed as previously reported [23]. Paraffin-embedded tissue sections were deparaffinized in xylene and rehydrated in graded series of ethanol followed by heat induced epitope retrieval in citrate buffer (PH 6.0). Rab18 expression were detected using anti-Rab18 polyclonal antibody (Proteintech Group).

### Statistical analysis

Data are presented as the mean ± standard deviation from at least three independent experiments. The two-tailed *t*-test was used to draw a comparison between groups. The null hypothesis was rejected at the 0.05 level.

## Results

### miR-30b/c directly targets Rab18 in human NSCLC cells

**Figure 1:**
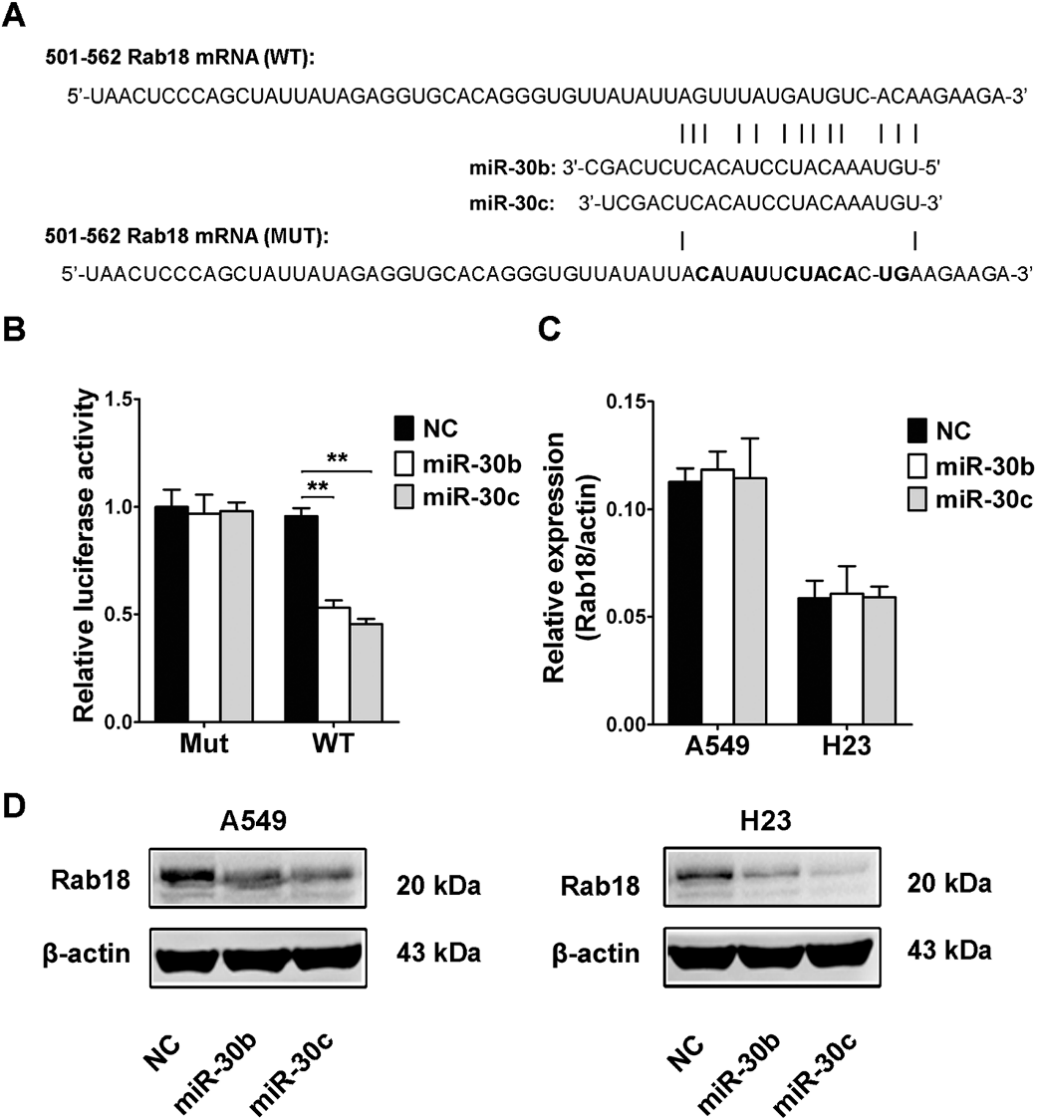
**miR-30b/c targets Rab18. (A)** Wild-type (WT) and mutant (Mut) of putative miR-30b and miR-30c targeting sequences in Rab18 mRNA. Mutant sequences were shown in bold type. **(B)** Analysis of luciferase activity. HEK293 cells were cotransfected with miR-30b or miR-30c mimics and negative control oligonucleotides, pRL-TK and firefly luciferase reporter plasmid containing putative miR-30b/c targeting sequences of Rab18. pRL-TK was cotransfected as an internal control to correct the differences in both transfection and harvest efficiencies. The firefly luciferase activity of each sample was normalized to the Renilla luciferase activity. The normalized luciferase activity control oligo nucleotides was set as relative luciferase activity 1 respectively. **(C,D)** Effects of miR-30b/c on the endogenous Rab18 expression levels. A549 and H23 cells were cotransfected with miR-30b or miR-30c mimics and negative control oligonucleotides. Forty-eight hours after transfection, cells were isolated, the mRNA levels **(C)** and protein levels **(D)** of Rab18 were analyzed by qRT-PCR and WB, respectively. (*P < 0.05, **P < 0.01, Student’s *t*-test).

In order to investigate the biological significance and its underlying mechanisms of the silenced miR-30 in NSCLC. As miRNAs are a group of post-transcriptional gene regulators which potentially play a critical role in tumorigenesis by regulating the expression of their target genes, the target genes of miR-30 that functioned in NSCLC pathogenesis was further analyzed. Newly published CLASH data in HEK293 cells provided us with the direct evidence for miRNA:mRNA pairing [24]. The CLASH data showed that both miR-30b and miR-30c targeted in coding DNA sequence of Rab18 which was associated with proliferation in hepatocellular carcinoma [23]. To confirm whether miR-30b/c regulated the expression of Rab18 gene, we first performed luciferase reporter assays in HEK293 cells. Our results showed that the reporter plasmid with wild-type targeting sequence of Rab18 mRNA caused a significant decrease in luciferase activity in cells transfected with miR-30b and miR-30c, whereas reporter plasmid with mutant sequence of Rab18 produced no change in luciferase activity (Figure 1A,B). Then, we explored whether the endogenous Rab18 in NSCLC cells was regulated similarly. A549 and H23 cells were transfected with miR-30b or miR-30c, and Rab18 protein levels and mRNA levels were examined by WB and qRT-PCR, respectively. Our results showed that Rab18 mRNA expression was not affected by miR-30b and miR-30c (Figure 1C), while the level of Rab18 protein was consistently and substantially down-regulated by miR-30b and miR-30c (Figure 1D). Taken together, our results demonstrated that Rab18 was a direct target of miR-30b/c in NSCLC cells.

### miR-30b/c are low-expressed and Rab18 is high-expressed in NSCLC tissue samples

**Figure 2:**
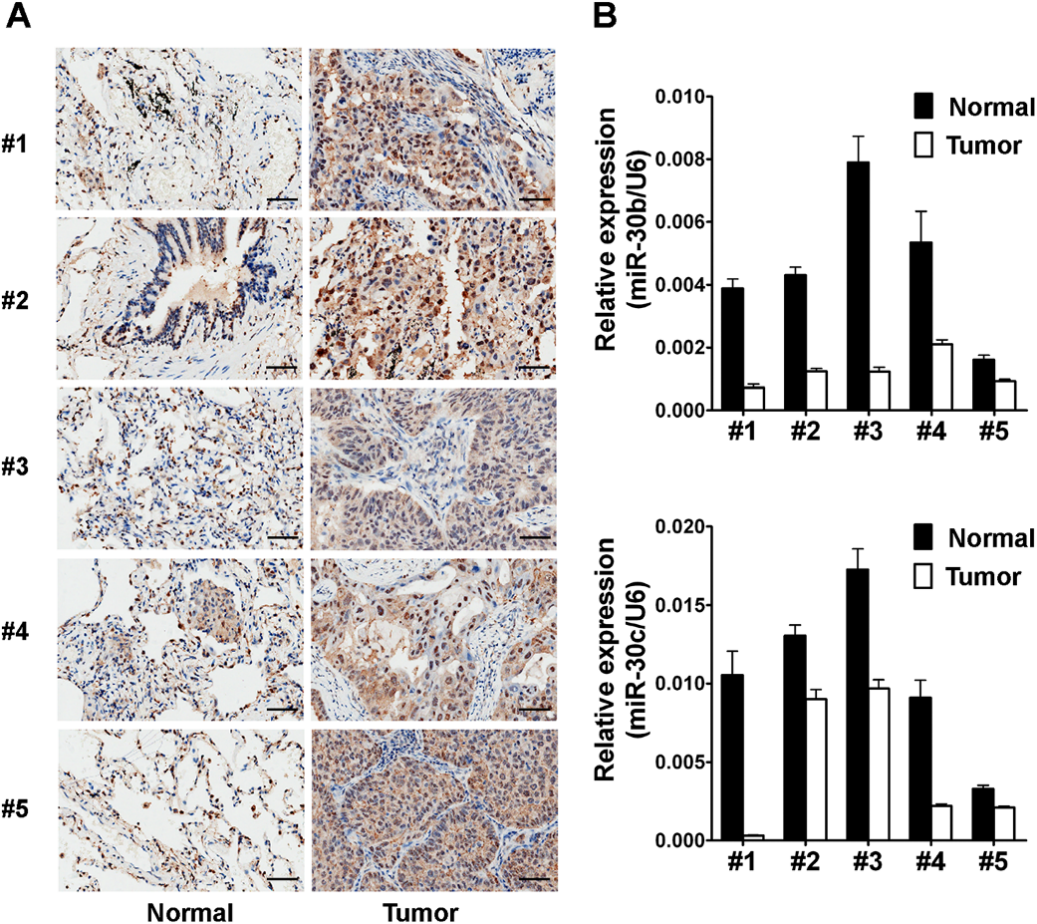
**Expression analyses of miR-30b/c and Rab18 in NSCLC tissues. (A)** IHC analysis of Rab18 expression in five pairs of lung cancer tissues and their corresponding non-tumor tissues, Brown signal in IHC was considered as positive staining for Rab18. Scale bars, 50 μm. **(B)** qRT-PCR analysis of miR-30b (up) and miR-30c (down) expression in five pairs of lung cancer tissues and their corresponding non-tumor tissues as indicated in **(A)**. The expressions of miR-30b/c were normalized to U6.

Prompted by our results that Rab18 was a direct target of miR-30b/c in NSCLC cells, we sought to investigate the association of miR-30b/c and Rab18 in NSCLC tissues. Rab18 protein expression of five pairs of clinical NSCLC and adjacent non-tomor tissues was analyzed by IHC. Our results showed that the protein levels of Rab18 were up-regulated in clinical NSCLC tissues compared with their adjacent non-tomor tissues (Figure 2A). Furthermore, we analyzed the expression of miR-30b/c in these five pairs of clinical NSCLC and adjacent non-tumor tissues by qRT-PCR and normalized to an endogenous control (U6 RNA). We found that miR-30b and miR-30c were down-regulated in these five pairs of clinical NSCLC tissues compared with their adjacent non-tomor tissues (Figure 2B). The results suggested that the reduced miR-30b/c expression and increased Rab18 protein expression were frequent events in human NSCLCs tissues.

### miR-30b/c inhibits NSCLC cell proliferation

**Figure 3:**
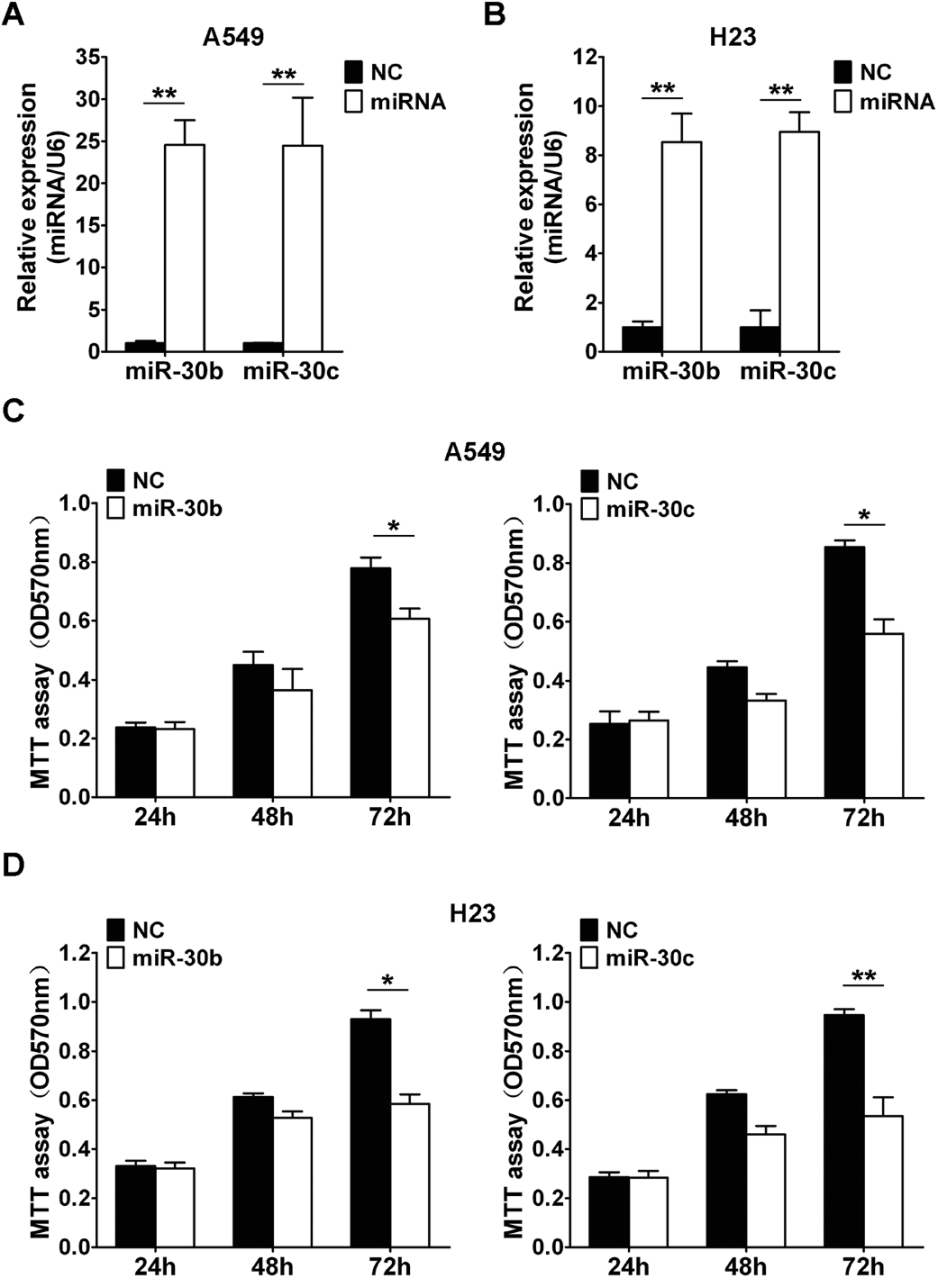
**Overexpression of miR-30b/c inhibits NSCLC cells growth** ***in vitro.*** **(A,B)** qRT-PCR analysis of miR-30b/c in A549 **(A)** and H23 **(B)** cells transfected with miR-30b/c mimics or negative control oligonucleotides. **(C,D)** The effects of miR-30b/c on A549 **(C)** and H23 **(D)** cells proliferation were determined by MTT assay at 24 h, 48 h, and 72 h after transfecting of miR-30b/c mimics or negative control oligonucleotides. (*P < 0.05, **P < 0.01, Student’s *t*-test).

In order to investigate the effect of miR-30b/c on NSCLC cell proliferation, miR-30b or miR-30c were transfected into A549 and H23 cells. qRT-PCR results determined that transfection of miR-30b or miR-30c increased their expressions in A549 (Figure 3A) and H23 (Figure 3B) cells. NSCLC cells proliferation was assessed by MTT assay. Our results showed that cellular proliferation gradually declined following transfection with miR-30b or miR-30c in A549 (Figure 3C) and H23 (Figure 3D) cells. Compared with the negative control, treatment of cells with miR-30b or miR-30c led to a decrease in NSCLC cell growth at 72 h, and the inhibitory efficiencies in A549 cells were 25.1% (*P* < 0.05) and 35.7% (*P* < 0.05), respectively, and the inhibitory efficiencies in H23 cells were 37.2% (*P* < 0.05) and 43.6% (*P* < 0.01), respectively. Taken together, these results demonstrated that miR-30b/c could inhibite the proliferation of NSCLC cells *in vitro*.

### miR-30b/c inhibits the proliferation of NSCLC cells via regulation of Rab18

**Figure 4:**
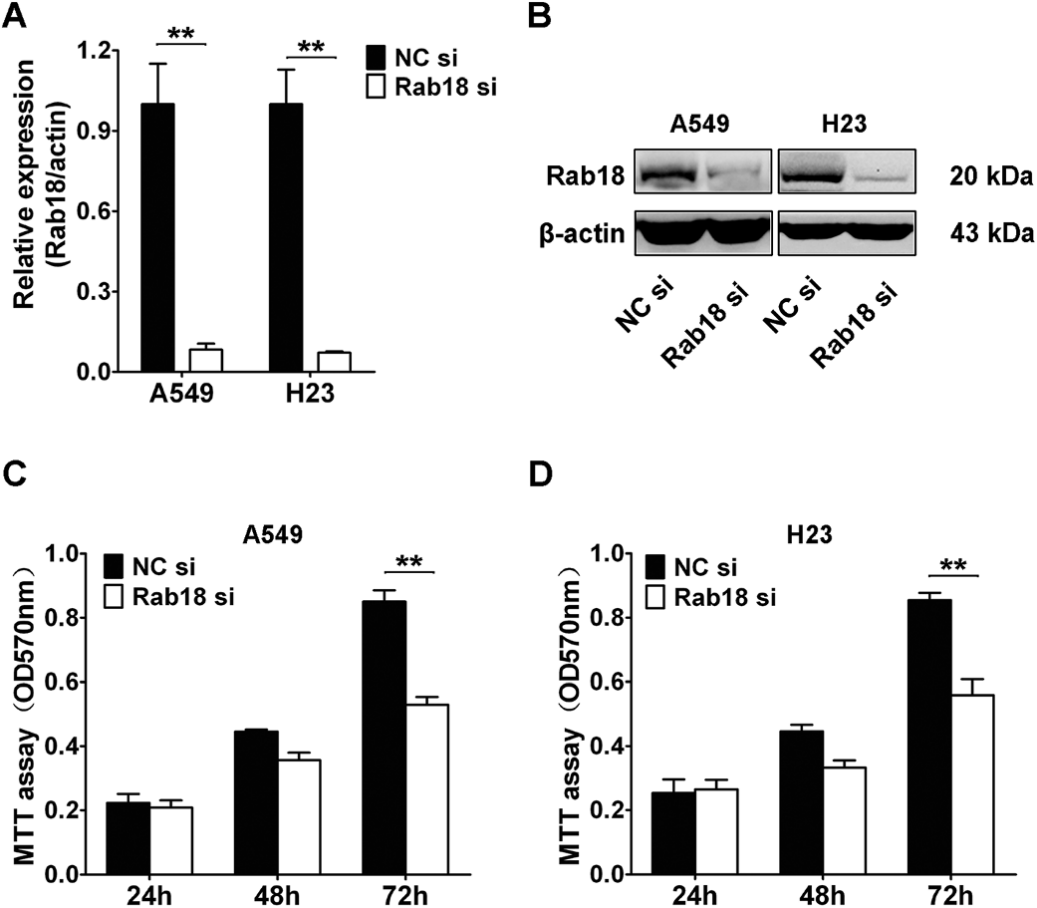
**Knockdown of Rab18 inhibits NSCLC cells growth** ***in vitro.*** **(A,B)** qRT-PCR **(A)** and Western blot analysis **(B)** were performed to determine the expression level of Rab18 after transfection of Rab18 siRNA and control siRNA in A549 and H23 cells. **(C, D)** The effects of Rab18 siRNA on A549 **(C)** and H23 **(D)** cells proliferation were determined by MTT assay at 24 h, 48 h, and 72 h after transfecting of Rab18 siRNA and control siRNA. (*P < 0.05, **P < 0.01, Student’s *t*-test).

Since overexpression of miR-30b/c suppressed the proliferation of NSCLC cells, and given that Rab18 is a direct target of miR-30b/c, we hypothesized that the inhibitory effect of miR-30b/c on NSCLC cell viability might be achieved via targeting Rab18. In order to investigate this hypothesis, we tested whether RNAi-mediated reduction in Rab18 levels influence the cell growth of NSCLC cells. Treatment of cells with Rab18 siRNA markedly decreased mRNA and protein levels of Rab18 in A549 and H23 cells (Figure 4A and B). MTT assay was performed to determine the effect of Rab18 siRNA on cell proliferation. The results showed that Rab18 siRNA treatment suppressed cell viability by 34% (*P* < 0.01) in A549 cells and 36.4% (*P* < 0.01) in H23 cells at 72 h, compared with control siRNA (Figure 4C). Our results agreed with the previously study showing that Rab18 was associated with hepatocellular carcinoma (HCC) proliferation. Our results demonstrated that down-regulation of Rab18 expression by miR-30b/c contributed, at least in part, to the suppression of the growth of NSCLC cells.

## Disscussion

Recently, attentions have focused on the role of miRNA in tumorigenesis. In this study, we focused on miR-30 which was decreased in several tumor types including NSCLC. Our results showed that Rab18 was one direct functional targets of miR-30b/c in NSCLC cells. Down-regulation of miR-30b/c and up-regulation of Rab18 protein levels were also found in NSCLC tissues compared to adjacent non-tumor tissues. Furthermore, ectopic overexpression of miR-30b/c blocked NSCLC cells proliferation *in vitro*.

The aberrant expression of miRNAs is associated with cancer progression including proliferation, migration, invasion and apoptosis. Deregulation of miRNAs such as miR-221, miR-222, miR-449a, miR-21, miR-205, miR-10b, miR-143 and miR-181a in NSCLC is a key factor underlying tumorigenesis [25]. Human miR-30 is down-regulated in several tumor types including NSCLC [20]. This suggests miR-30 is a potential tumor suppressor. These findings prompted us to investigate the regulation of miR-30 in NSCLC cells. Recent studies showed that miR-30a regulated growth of breast cancer cells [26], down-regulation of miR-30 maintained self-renewal and inhibited apoptosis in breast tumor-initiating cells [27], miR-30 regulated B-Myb expression during cellular senescence [28]. However, the role of miR-30 in cancers especially in NSCLC is not very much known. In this study, we confirmed that oncogene Rab18 was directly targeted by miR-30b/c in NSCLC cells. Decreased miR-30b/c and increased Rab18 protein expression were also found in NSCLC tissues, which suggested that Rab18 was regulated by miR-30b/c in human NSCLC tissues. Human miR-30 family including miR-30a, miR-30b, miR-30c, miR-30d and miR-30e have the samilar sequence. Whether other miR-30 family have the samilar function like miR-30b and miR-30c in NSCLC or other cancer cells remain to be investigated.

Rabs, small G proteins belonging to the Ras superfamily, are regulators of vesicular transport in both exocytic and endocytic pathways in eukaryotic cells [29]. Emerging evidences have revealed the association between dysfunction of the Rab18 and multiple human diseases including cancer [29–31]. It had been reported that Rab18 was involved in the lipogenesis of 3T3-L1 adipocytes [23]. Loss-of-function mutations in Rab18 caused Warburg Micro syndrome [32]. Moreover, Rab18 acted as a novel tumor antigen in medulloblastoma and HCC [23, 33]. However, the expression of Rab18 is less well known in human cancers especially in NSCLC. In this study, we first reported that Rab18 protein levels were highly expressed in NSCLC tissues compared to matched adjacent non-tumor tissues. To investigate the underlying mechanism of up-regulation of Rab18 protein levels in NSCLC. miRNA-binding sites analysis revealed that Rab18 was one direct functional target of miR-30b/c in NSCLC cells. Transfection of miR-30b/c mimics into NSCLC cells led to a significant Rab18 decrease at protein levels but not mRNA levels and inhibition of cellular proliferation. Furthermore, silencing Rab18 expression by siRNA in NSCLC cells also led to inhibition of cellular proliferation. These findings support the hypothesis that decreased expression of Rab18 by miR-30b/c accounts for the suppression of cellular proliferation in NSCLC. Our results agreed with the previous study in HCC which showed that Rab18 was directly targeted and down-regulated at protein levels by miR-429 in HCC cells, but whether the expression of Rab18 was also regulated at transcriptional levels in NSCLC cells just like that by hepatitis B virus X protein stimulation in HCC cells remain to be investigated [23].

## Conclusions

Taken together, we demonstrate that miR-30b/c is down-regulated in NSCLC tissues. Overexpression of miR-30b/c directly down-regulates Rab18 and inhibits NSCLC cell proliferation. These data indicate that miR-30b/c could serve as a tumor suppressor gene involved in NSCLC pathogenesis.

## Abbreviations

NSCLC: Non-small cell lung cancer
mRNA: Messenger RNA
qRT-PCR: Quantitative reverse transcription-polymerase chain reaction
miRNA: microRNA
FBS: Fetal bovine serum.
